# Preoperative Anaemia and Associated Postoperative Outcomes in Noncardiac Surgery Patients in Central Region of Ghana

**DOI:** 10.1155/2017/7410960

**Published:** 2017-12-11

**Authors:** Gladys Amponsah, Alice Charwudzi

**Affiliations:** ^1^Department of Anaesthesia and Pain Management, School of Medical Sciences, University of Cape Coast, Cape Coast, Ghana; ^2^Department of Chemical Pathology, School of Medical Sciences, University of Cape Coast, Cape Coast, Ghana

## Abstract

**Introduction:**

Several studies suggest that preoperative anaemia (PA) is associated with adverse postoperative outcomes, but little is known about these outcomes in the Central Region of Ghana. This study aims to determine the prevalence of PA among noncardiac surgical patients and its implications for their postoperative outcomes.

**Methods:**

This study was designed as an observational study; data including demographics and clinical and laboratory results were collected from the patients' records and through interviews.

**Results:**

A total of 893 inpatient surgical cases undergoing elective and emergency operations, aged 15 years and above with mean age of 44.2 ± 17.0 yrs, were enrolled. The prevalence of PA was 54.3%, mostly microcytic with or without hypochromia (57.2%). The prevalence was higher in females than males (*p* ≤ 0.001). Preoperative anaemia was significantly associated with prolonged length of hospital stay (OR: 2.12 (95% CI: 1.49–3.10)). Allogeneic blood transfusion significantly prolonged the length of hospital stay (OR 4.48 (95% CI: 2.67–7.51)). 15.5% of the anaemic patients received oral iron supplements compared to 2.2% of nonanaemic patients (*p* ≤ 0.001).

**Conclusion:**

Preoperative anaemia is common among noncardiac surgical patients. It is independently and significantly associated with prolonged hospital stay leading to the use of increased healthcare resources. It is also the main predictor for perioperative allogeneic blood transfusions and the use of haematinics.

## 1. Introduction

Anaemia is an important public health concern that has negative impact on an individual's health as well as the economic potential of the population [1]. The prevalence of anaemia depends on age, sex, and associated comorbidities such as diabetes, hypertension, and inflammatory conditions [2].

Preoperative anaemia is not an uncommon finding, and depending on the cohort being investigated, the prevalence can be as high as 75% [3]. Preoperative anaemia increases the risk of oxygen depletion, thereby increasing the risk of unfavourable postoperative outcomes [3]. These patients are either given large amounts of red blood cell transfusions depending on the severity of the anaemia [4] or in nonurgent cases necessitating the postponement of surgery [5] till the haemoglobin concentration stabilises or normalises.

Allogeneic blood transfusion is independently associated with increased risk of infection and other adverse postoperative outcomes, especially in countries with low or medium human development index [6, 7]. Furthermore, the treatment of preoperative anaemia with RBC transfusions is more costly as compared to correcting anaemia with haematinics like iron, vitamin B_12_, folate, or erythropoietin substitutes preoperatively [8]. Blood transfusion therapy may also increase the risk of transfusion transmissible infections (TTIs) and alloimmunization in a low-to-middle income country such as Ghana. The estimated TTI risk for HIV, HBV, and HCV from a unit of transfused blood in sub-Saharan Africa is 1, 4.3, and 2.5 infections per 1000 units, respectively, based on a mathematical projection [9]. These three microbiological agents and syphilis are the ones routinely screened in Ghana.

Malaria, a multiorgan systemic disease, can cause and aggravate anaemia, constituting an additional risk in surgical procedures. Studies have demonstrated high rates of morbidity and mortality after surgery in anaemic patients with *Plasmodium falciparum* associated malarial disease [10]. In addition to anaemia, other confounding factors such as the comorbid conditions can also impede the postoperative progress of the patient [8].

Preoperative haemoglobin levels are checked before surgery in most patients in Ghana. As far as we are aware, no studies have explored the implications of preoperative anaemia on postoperative outcomes in Ghana. This study was therefore carried out to determine the prevalence of preoperative anaemia in unselected noncardiac surgery patients and their outcomes following surgery in the Central Region of Ghana.

## 2. Materials and Methods

This study was designed as an observational study and was carried out from June 2015 to July 2016 in two selected hospitals in the Central Region of Ghana. Ethical approval for the study was obtained from the Institutional Review Board of the University of Cape Coast (approval reference: UCC/IRB/3/1). Written permission was also sought from the Management of the Cape Coast Teaching Hospital, Cape Coast, and Saint Luke's Catholic Hospital, Apam. Written informed consent was obtained from individual patients or their guardians. Eligible patients aged 15 and above had a preoperative assessment within 40 days prior to surgery with at least one preoperative haemoglobin (Hb) level to undergo elective or emergency surgery were recruited. Exclusion criteria included planned day-case surgery and obstetric procedures [3, 7, 8]. Data were collected using the patient's medical record, operation logbooks, anaesthetic records, nurses review notes, and interviews/interaction with surgeons on admission and discharge of the patient. Data were entered into an Excel 2010 sheet anonymously. Patients who were on admission for more than 15 days before surgery, patients who were on admission for more than 30 days after surgery and those with incomplete data such as missingdate of discharge were excluded from the data analysed. Participation did not require any additional visits or assessments. When data collection was not completed before discharge, the participant's medical record was retrieved from the medical records unit. The administration of RBC and haematinics followed the selected hospitals' protocols. A review of the autologous donation records and interview with the blood bank organiser were used to obtain data on autologous donation and the cost of processing a unit of whole blood. Relatives of patients who brought in replacement donors were also interviewed on the cost of presenting a single possible donor.

### 2.1. Data Collection

Data were collected on patients' characteristics (sex and age), date of admission, date of surgery, date of discharge, preoperative full blood count result, type of anaesthesia (general or regional), type of surgery done, duration of surgery, comorbidity, sickling status, presence of malaria parasites, date of inhospital mortality, complications before discharge, and date of RBC transfusion. The primary outcome measured was preoperative anaemia. Secondary outcomes were postoperative adverse events resulting in complications before discharge, prolongation of hospitalization, the prevalence of perioperative allogeneic blood transfused, and inhospital mortality. Patients were followed up until they were discharged from the hospital. Complete patient confidentiality was maintained.

### 2.2. Definitions

Preoperative haemoglobin (Hb) levels were the last Hb estimated within 40 days prior to the index operation; majority of the participants 773 (86.6%) had their last Hb estimated within two weeks prior to the index surgery. The World Health Organization (WHO) criteria for anaemia (women 12.0 g/dl and men 13.0 g/dl) and its subclassifications (nonpregnant women 15 years of age and above: mild: 11.0–11.9 g/dl, moderate: 8.0–10.9 g/dl, severe: lower than 8.0 g/dl; men 15 years of age and above: mild: 11.0–12.9 g/dl, moderate: 8.0–10.9 g/dl, severe: lower than 8.0 g/dl) were used [11]. Morphological examination of peripheral blood film for anaemia classification was not routinely performed at the selected centres. Hence, anaemia was classified based on mean corpuscular volume (MCV) and/or mean corpuscular haemoglobin (MCH) into (i) normocytic normochromic types (MCV ≥ 80 but <100 fl; MCH ≥ 27 pg): primary aetiology likely to be anaemia of chronic disease, (ii) microcytic (MCV < 80 fl) and/or hypochromic types (MCH < 27 pg): possible iron deficiency, and (iii) macrocytic types (MCV ≥ 100 fl): possible folate or B_12_ deficiency. The surgical procedures (grade of surgery) were classified using the BUPA Schedule of Procedures [12].

Postoperative outcomes included inhospital mortality, prolonged length of hospital stay (LOS) after surgery, and the development of postoperative infection or other complications such as cardiogenic shock before discharge and perioperative blood transfusion. Prolonged LOS was categorized as the proportion of patients with hospital stay greater than the 75th percentile (8.0 days), as used by other studies [13, 14].

### 2.3. Statistical Analysis

Descriptive statistical analysis: continuous data were presented as mean, standard deviation (SD), or minimum and maximum values; categorical data were presented as the number and percentage of individuals in each category; and Pearson's chi-square test was used to compare the proportions between two groups.

Binary logistic regression models were performed using adjusted odds ratios (ORadj) to assess the independent effects of preoperative anaemia on length of hospital stay, postoperative complications, inhospital mortality, and perioperative RBC transfusion. Only factors remaining statistically significant (*p* < 0.05) from the univariate analysis (sex, surgical specialty, and comorbid conditions) and factors that had been theoretically proven to have effects (i.e., age, grade of surgery, and cancer) were used in the final model as covariates. Patients with severe and moderate anaemia were analysed together due to small numbers for the severely anaemic.

Analysis was performed with IBM SPSS statistical package version 21.0, USA.

## 3. Results

### 3.1. Patient Characteristics

In all, 953 eligible patients were enrolled in the study. 60 (6.3%) patients were excluded. The excluded patients included patients whose period of admission days was outside those used in the analysis (48), patients with incomplete data (6), and patients with miscellaneous reasons such as high Hb (6). Hence, after data cleaning, 781 (87.5%) patients from a tertiary hospital and 112 (12.5%) from a mission hospital were included in the analysis. The details of the patient's characteristics are shown in Tables 1 and 2.

433 (48.5%) patients had general anaesthesia (GA), 434 (48.6%) had subarachnoid spinal block (SAB), 10 (1.1%) had SAB and GA, and 16 (1.8%) had other anaesthesias such as local infiltration. There were a total of 624 (69.9%) elective cases out of which 342 (54.8%) were anaemic, and 269 (30.1%) emergency cases out of which 143 (53.2%) were anaemic. The overall median operating time (interquartile) was 76.0 minutes (50.0,111.0 minutes).

### 3.2. Prevalence of Preoperative Anaemia

Patients with preoperative anaemia accounted for 54.3% of the study population, out of which 209 (23.4%) presented with mild anaemia, 226 (25.3%) with moderate anaemia, and 50 (5.6%) with severe anaemia. The sex distribution, the mean Hb, the severity, and the classification of the anaemia as well as surgical specialty are shown in Table 1. Age did not differ among the groups (*p*=0.326). The significant association of anaemia and comorbidity is shown in Table 2. Hypertension was the most common comorbidity associated with anaemic patients.

When excluded data were included in the analysis, prevalence of preoperative anaemia was 55.2%; it did not skew the anaemia prevalence.

#### 3.2.1. Age and Sex Stratification of Anaemic Patients

The anaemia was *more* predominant among the 15–49 years group than the 50–90 years (*p*=0.003). Using this stratification, comorbidity, severity, and classification of the anaemia are shown in Table 3.

Elderly patients (65 years and above) constituted 133 (14.9%) of the study population. The elderly group recorded the highest prevalence of preoperative anaemia 82 (61.7%) compared to those below 65 years (53.0%) (*p*=0.065). 44 of 82 (53.7%) had mild anaemia with only 8 (9.8%) presenting with severe anaemia. The anaemia type was mainly normocytic normochromic (45/70; 64.3%) as compared to anaemic patients (167/358; 46.6%) below 65 years (*p*=0.021). Also, 36 (43.9%) of the anaemic elderly patients had comorbid conditions compared to 13 (25.5%) for the nonanaemic patients (*p*=0.027).

### 3.3. Postoperative Outcomes

Evaluation of postoperative outcomes included the length of hospital stay after surgery, postoperative complications before discharge, and inhospital mortality.

- Length of hospital stay: preoperative anaemia patients had a significantly higher mean length of hospital stay (6.5 days; range: 1–29 days) than nonanaemic patients (4.8 days; range: 1–27 days) (*p* ≤ 0.001). This probability continued to increase with decreasing Hb concentration in the preoperative anaemia group. The mean in days was 5.9 for mildly anaemic, 6.7 for the moderately anaemic, and 8.2 for the severely anaemic patients. After logistic regression analysis adjusting for potential confounders (statistically significant baseline characteristics and those with theoretically proven effects), preoperative anaemia remained independently and significantly associated with increased hospital length stay (Table 4).
- Complications before discharge: the details of the complications before discharge are shown in Table 5. There was no difference among groups regarding the composite complications (*p*=0.101).
- Inhospital mortality: mortality data were available for the tertiary hospital (781) cases only. *There was no difference among groups regarding inhospital mortality* (mortality for anaemic group 8/398 (2.0%) versus nonanaemic 4/383 (1.0%), (*p*=0.273). Only 12 (1.3%) inhospital mortalities were recorded. Mortality occurred between 0 and 28 days after surgery. Most of the mortality (66.7%) occurred among emergency general surgery cases.

### 3.4. Effect of Perioperative Red Cells Transfusions on Preoperative Anaemia

Perioperative anaemia was treated mainly with allogeneic RBC transfusion followed by haematinics. Overall, 172 (19.3%) patients received a total of 347 units of allogeneic RBC transfusion, 73 (8.2%) patients received two units of RBCs, followed by 1 unit for 55 (6.2%) patients, with the remaining patients receiving between 3 and 5 units. Overall, patients with preoperative anaemia received more perioperative transfusion than nonanaemic patients (30.7% versus 5.6%, *p* ≤ 0.001; OR 7.42, 95% CI (4.67–11.79)). This difference was also consistent with preoperative transfusion (12.8% versus 0.7%, *p* ≤ 0.001), intraoperative transfusion (10.7% versus 1.5%, *p* ≤ 0.001), and postoperative transfusion (16.7% versus 4.2%, *p* ≤ 0.001), with some patients receiving transfusion in more than one phase of the perioperative phases.

By binary logistic regression analysis, independent predictors of RBC transfusion were severity of preoperative anaemia, severe and moderate anaemia (OR 16.83, 95% CI (9.16–30.89), *p* ≤ 0.001), and comorbid conditions (OR 2.57, 95% CI (1.46–4.52), *p*=0.001) after adjusting for confounding factors; non-RBC transfused group was used as a reference. Mild anaemia was not a predictor for transfusion (OR 1.29, 95% CI (0.62–2.65), *p*=0.495). After adjusting for these confounding factors including age and sex, perioperative RBC transfusion significantly and independently was associated with prolonged length of hospital stay. Severe and moderate anaemia became a stronger determinant for RBC transfusion (*p* ≤ 0.001). Comorbidity still remained a significant determinant (OR 2.22, 95% CI (1.29–3.82), *p*=0.004) of RBC transfusion. There was no significant association between inhospital mortality with perioperative RBC transfusion after adjusting for significant confounding factors as shown in Table 6.

For preoperative transfusion, 61/65 (93.8%) of patients were haemo transfused within 2 weeks to the surgery. Additionally, 70/98 (71.4%) of patients received their postoperative transfusion within 2 days after surgery. Some patients (14/65; 21.5%) were transfused before hospital admission. The blood bank recorded two autologous predonations during the study period. However, the patients involved were not transfused; hence, all transfusions were allogeneic.

#### 3.4.1. Cost Implications of Allogeneic RBC Transfusion

Data available from the blood bank records at the tertiary hospital and interview with patients indicated an approximate cost of 160.0 Ghanaian cedis (approximately 38.0 USD) for obtaining and processing one unit of replacement donation.

### 3.5. Haematinics

180 (20.2%) patients were treated with perioperative haematinics: 151 (31.1%) for anaemic as compared to 28 (6.9%) for nonanaemic patients (*p* ≤ 0.001). Preoperative haematinics use among anaemic versus nonanaemic patients was 12.0% versus 1.2% (*p* ≤ 0.001). In addition, 122 (25.2%) of the anaemic patients received haematinics postoperatively compared with 24 (5.9%) of the patients without anaemia (*p* ≤ 0.001), with some patients receiving multiple modes of treatment. Oral iron was frequently used for perioperative anaemia treatment as shown in Table 7.

### 3.6. Exclusion

Excluded data did not skew the overall data, when analysed as shown in Table 8.

### 3.7. Other Findings

#### 3.7.1. Postoperative Malaria

Postoperative malaria parasitaemia data were available for 423 patients. Positive patients who were symptomatic were treated before surgery. The prevalence rate of malaria parasitaemia in the entire study population increased from 2.5% preoperatively to 3.5% postoperatively (*p* ≤ 0.001). The prevalence rate increased from 3.2% before surgery to 4.2% (*p* ≤ 0.001) after surgery among the anaemic patients and from 1.8% to 3.4% among the nonanaemic patients (*p* ≤ 0.001).

## 4. Discussion

A high prevalence of preoperative anaemia (approximately 54%) in unselected noncardiac surgical patients was observed in our study, but most had moderate anaemia. Overall, 57% of anaemic admissions had microcytic and/or hypochromic indices, consistent with possible iron deficiency, but normocytic normochromic indices were prevalent in the elderly (65 years and above) together with a significantly higher comorbidities, indicating a possible anaemia of chronic disease or inflammation. The preoperative anaemia resulted in prolonged postoperative hospital stay when compared with the nonanaemic patients. Composite complications and inhospital mortality were more frequent among the anaemic patients though not statistically significant. In this study, about 31% of preoperative anaemia patients received perioperative transfusion with an overall mean of two units per patient. The independent determinants for blood transfusion were the severity of the preoperative anaemia and comorbid condition. Perioperative transfusion was significantly associated with poor postoperative outcomes.

This study is in agreement with other studies showing high levels of preoperative anaemia among unselected surgical patients [8] and among elective orthopaedic patients [3]. However, the high prevalence rate of 54% despite the preoperative transfusion and haematinics use contradicts previous studies of about 29% [8], 25% [15], and 14% [3]. Approximately, 47% prevalence rate was reported among nonpregnant Ghanaian women of reproductive age (15–49 years) in the Central Region of Ghana using the WHO criteria [16]. But our findings showed a higher prevalence rate of about 66% among women in the reproductive age group even though women formed 50.8% of the study population. Olayemi and colleagues reported approximately 52% anaemia among apparently healthy young Nigerian adults [17].

Saleh and colleagues [18] reported 23% possible iron deficiency among 1142 elective major joint arthroplasty patients aged 15–91 years using hypochromasia as an index. Our study recorded approximately 57% anaemic patients with microcytosis and/or hypochromasia. About 64% of females in the reproductive age group had microcytic and/or hypochromic indices. This is in line with the high prevalence of iron deficiency among black Africans including Ghanaians. A prevalence of 32.0% was reported among Ghanaian women living in Germany [19], 41–63% iron deficiency among Ivoirians women [20], and 39% IDA among Nigerians women aged 16–45 years [21]. Thalassaemia is equally prevalent in Ghanaians, alpha-thalassaemia is about 34% [19] and beta-thalassaemia is about 5% [22], but could not be differentiated due to restriction on confirmatory investigations at the selected centres. Anaemia of chronic diseases could not also be excluded.

61% of the elderly patients had anaemia which was mostly mild with normocytic normochromic indices. They also had significant comorbidities suggesting possible anaemia of chronic disease or inflammation.

Regarding outcomes, preoperative anaemia remained an independent risk factor for prolonged hospital stay after adjusting for potential confounders. Some observational studies in noncardiac surgical patients emphasize this association [9, 23].

The overall composite complication before discharge was 13.4%. Although comorbidity was significantly greater in preoperative anaemia patients, the anaemia did not significantly modify the composite complication. However, other studies have found anaemia to be an independent predictor for postoperative complications [6, 8]. This is probably due to the inability of this study to capture complications such as renal insufficiency. The short duration in which the complications were recorded may be a contributing factor.

Our study did not show a significant association between preoperative anaemia and inhospital mortality. This agrees with the findings of Greenky and colleagues [24] and Munoz and colleagues [25]. However, Baron et al. [8] found preoperative anaemia to be significantly and independently associated with inhospital mortality. Saager and colleagues [15] found anaemia to be a weak independent predictor of 30-day postoperative mortality.

Our study is also in agreement with previous studies showing preoperative anaemia to be an independent and strong predictor for increased perioperative RBC transfusion [3, 7]. The result of this study suggests that allogeneic RBC transfusion is significantly associated with prolonged length of hospital stay, higher complications, and higher mortality. After correcting for patients characteristics, prolonged length of hospital stay remained independently and significantly associated with allogeneic RBC transfusion [3].

This study recorded a relatively low perioperative transfusion rates compared to the high prevalence of preoperative anaemia. This may be reflective of the type of surgery, the sample size, and amounts of blood loss (which was not captured). Some patients could not get replacement donors and had to be discharged with fewer units of blood transfused or without being transfused when the Hb level was not life-threatening. In the hospitals where the research was carried out, voluntary blood donation is not common; hence, patients have to rely on replacement donation which can be difficult to obtain. Transfusion attracts an extra financial burden on the patient which most cannot afford.

Despite recent recommendations advising investigating the cause of anaemia and treating it preoperatively to minimise transfusion requirements in elective surgical patients, the percentage of anaemic patients treated preoperatively was very low, about 12%. Preoperative oral or parenteral iron or postoperative parenteral iron with or without erythropoietin-stimulating therapy is useful for the management of perioperative anaemia [8, 18].

Traumatic events like tissue damage, blood loss, and surgery trigger cascades of inflammatory mediators to cause immunodepression with increased risk to develop malaria parasitaemia [26]. In a study among traumatized land mine and war victims in Cambodia [27], about 33% of asymptomatic malaria carriers developed posttrauma and postsurgery symptomatic malaria. Our study showed a significant increased prevalence rate of postoperative malaria parasitaemia in agreement with these findings [26, 27]. Ghana being an endemic area for malaria, the immunosuppression due to surgery predisposes patients to malaria parasitaemia. However, very few of the patients in this study were symptomatic postoperatively. Our study, however, contradicts the study by Takongmo and colleagues [28], which showed that the development of postoperative malaria was not significantly associated with the surgical trauma.

## 5. Limitations

About 1.5% of the preoperative Hb concentrations were obtained more than 28 days prior to the surgery and may not have represented the actual Hb level at the time of surgery. Approximately 6.8% of the patients were on preoperative haematinics, and 7.3% were transfused prior to the surgery; however, there was no repeat of the Hb levels for a number of the patients after the initial assessment.

Postoperative Hb levels were not available for most patients; hence, their effects could not be analysed.

Also, anaemia was classified based on only mean corpuscular volume (MCV) and mean corpuscular haemoglobin (MCH); increased reticulocyte count due to compensatory response to anaemia and/or the preoperative haematinics administered to some patients can spuriously raise the MCV value masking microcytosis. Confirmatory tests such as ferritin or soluble transferrin receptor measurement for the diagnosis of iron deficiency are not routinely performed at the selected centres. Additionally, data on renal and liver function were not uniformly available for most patients and hence not captured.

## 6. Conclusion

Preoperative anaemia is common among noncardiac surgery patients. It is independently and significantly associated with prolonged hospital stay leading to increased healthcare resource use. It is also the main predictor for perioperative allogeneic blood transfusions and the use of haematinics.

This study has potential implications for improving the perioperative management of noncardiac surgery patients in the Central Region and Ghana as a whole. It will enhance adequate preparation and comprehensive care to minimise known complications associated with preoperative anaemia. It will also serve as a pilot for a wider national study on postoperative outcomes among noncardiac surgery patients.

## Figures and Tables

**Table 1:** Patient baseline characteristics.

Parameter	Total (*n* = 893)	Preoperative anaemia (*n* = 485)	No preoperative anaemia (*n* = 408)	*p* value
*Age, mean (range)*	44.2 (16–90)	45.3 (16–90)	43.0 (16–85)	0.326
15–49 years	565 (63.3%)	297 (61.2%)	268 (65.7%)	0.370
50 years and above	328 (36.7%)	188 (38.8%)	140 (34.3%)	—
*Sex, n (%)*				<0.001
Females	454 (50.8%)	293 (60.4%)	161 (39.5%)	—
Males	439 (49.2%)	192 (39.6%)	247 (60.5%)	—
*Preoperative Hb concentration*				
Mean Hb ± SD	12.0 + 2.5 g/dl	10.3 ± 1.8 g/dl	14.1 ± 1.3 g/dl	<0.001
Classification of anaemia^∗^	*n* = 804	*n* = 428	*n* = 376	<0.001
Microcytic and/or hypochromatic	396 (49.3%)	245 (57.2%)	151 (40.2%)	—
Normocytic normochromic	400 (49.7%)	179 (41.8%)	221 (58.8%)	—
Macrocytic	8 (1.0%)	4 (0.9%)	4 (1.1%)	—
*Grade of surgery*, *n *(%)^∗∗^				0.360
Minor	31 (3.5%)	17 (3.5%)	14 (3.4%)	—
Intermediate	180 (20.2%)	103 (21.2%)	77 (18.9%)	—
Major	672 (75.3%)	360 (74.2%)	312 (76.5%)	—
Complex major	10 (1.1%)	5 (1.0%)	5 (1.2%)	—
*Surgical specialty*, *n (%)*				<0.001
General surgery	583 (65.3%)	290 (59.8%)	293 (71.8%)	—
Gynaecology	147 (16.5%)	119 (24.5%)	28 (6.9%)	—
Orthopaedics	49 (5.5%)	19 (3.9%)	30 (7.4%)	—
Urology	51 (5.7%)	26 (5.4%)	25 (6.1%)	—
Plastics	24 (2.7%)	12 (2.5%)	12 (2.9%)	—
Ear, nose, and throat (ENT)	24 (2.7%)	13 (2.7%)	11 (2.7%)	—
Maxillofacial	15 (1.7%)	6 (1.2%)	9 (2.2%)	—

Results are shown as mean (range), mean (±SD), and frequency (percentage); % = percentage of patients; Hb = haemoglobin; ^∗^data on MCV and MCH were available on 804 (90.0%) patients; ^∗∗^grade of surgery was classified using the BUPA Schedule of Procedures [12].

**Table 2:** Patient baseline characteristics.

Parameters	Total (*N* = 893)	Preoperative anaemia (*n* = 485)	No preoperative anaemia (*n* = 408)	*p* value
*Comorbidities*				0.028
Hypertension	68 (7.6%)	40 (8.2%)	28 (6.8%)	—
Diabetes mellitus	28 (3.1%)	22 (4.5%)	6 (1.5%)	—
Diabetes mellitus + hypertension	27 (3.0%)	22 (4.5%)	5 (1.2%)	—
HIV	9 (1.0%)	5 (1.0%)	4 (1.0%)	—
Asthma	6 (0.7%)	3 (0.6%)	3 (0.7%)	—
Hepatitis B	5 (0.6%)	3 (0.6%)	2 (0.5%)	—
Diabetes mellitus + other conditions^∗^	4 (0.4%)	2 (0.4%)	2 (0.5%)	—
Others^‡^	5 (0.6%)	3 (0.6%)	2 (0.5%)	—
*Cancer*	50 (5.6%)	31 (6.4%)	19 (4.7%)	0.261
*Sickling status (N = 497)* ^∗∗^				0.101
Positive	52 (10.5%)	33/262 (12.6%)	19/235 (8.1%)	—
Negative	445 (89.5%)	229/262 (87.4%)	216/235 (91.9%)	—
	
*Preoperative malaria parasites (N = 600)* ^∗∗^				0.456
Positive	16 (2.7%)	10/320 (3.1%)	6/280 (2.1%)	—
Negative	584 (97.3%)	310/320 (96.9%)	274/280 (97.9%)	—

Results are shown as frequency (percentage); ^∗^diabetes mellitus with or without hypertension + (peptic ulcer disease or asthma or pneumonia); ^‡^alcoholic liver disease, chronic kidney disease, and chronic skin ulcer; ^∗∗^sickling and preoperative malaria results were available for 497 and 600 patients, respectively.

**Table 3:** Age and sex stratification.

	Males						Females					
	15–49 yrs (*N* = 245)			≥50 yrs (*N* = 194)			15–49 yrs (*N* = 320)			≥50 yrs (*N* = 134)		
Parameters	Anaemic	Nonanaemic	*p* value	Anaemic	Nonanaemic	*p* value	Anaemic	Nonanaemic	*p* value	Anaemic	Nonanaemic	*p* value
*Preoperative anaemia*	86 (35.1%)	159 (64.9%)	<0.001	106 (54.6%)	88 (45.4%)	0.238	211 (65.9%)	109 (34.1%)	<0.001	82 (61.2%)	52 (38.8%)	<0.001
*Comorbidities*	7 (8.1%)	8 (5.0%)	0.216	39 (37.5%)	16 (18.2%)	0.003	22 (10.4%)	12 (11.0%)	0.957	32 (39.0%)	16 (30.7%)	0.331
*Mean Hb (g/dl) ± SD*	11.0 ± 1.5	14.9 ± 1.4	<0.001	10.9 ± 1.7	14.4 ± 1.2	<0.001	9.8 ± 1.9	13.1 ± 0.9	<0.001	9.9 ± 1.5	13.0 ± 0.8	<0.001
*Severity of anaemia*	—	—	<0.001	—	—	<0.001	—	—	<0.001	—	—	<0.001
Mild	48 (55.8%)	0.0%	—	61 (57.5%)	0.0%	—	70 (33.2%)	0.0%	—	30 (36.6%)	0.0%	—
Moderate	34 (38.1%)	0.0%	—	41 (38.7%)	0.0%	—	109 (51.7%)	0.0%	—	42 (51.2%)	0.0%	—
Severe	4 (4.8%)	0.0%	—	4 (3.8%)	0.0%	—	32 (15.2%)	0.0%	—	10 (12.2%)	0.0%	—
*Classification of anaemia* ^∗^	*n* = 77	*n* = 145	0.060	*n* = 94	*n* = 81	0.114	*n* = 184	*n* = 102	0.003	*n* = 73	*n* = 48	0.049
Microcytic and/or hypochromic	46 (59.7%)	64 (44.1%)	—	40 (42.6%)	23 (28.4%)	—	118 (64.1%)	48 (47.1%)	—	40 (54.8%)	16 (33.3%)	—
Normocytic normochromic	31 (40.3%)	81 (55.9%)	—	52 (55.3%)	54 (66.7%)	—	66 (35.9%)	54 (52.9%)	—	31 (42.5%)	32 (66.7%)	—
Macrocytic	0 (0.0)	0 (0.0%)	—	2 (2.1%)	4 (4.9%)	—	0 (0.0%)	0 (0.0%)	—	2 (2.7%)	0 (0.0%)	—

Results are shown as mean ± SD and frequency (percentage); ^∗^Data on MCV and MCH were not available for all patients.

**Table 4:** Effects of preoperative anaemia on postoperative outcomes.

Outcome	Preoperative anaemia	
	OR_unadj_ (95% CI); *p* value	OR_adj−1_ (95% CI); *p* value
Prolonged LOS	1.72 (1.23–2.41); *p*=0.001	2.12 (1.49–3.10); *p* ≤ 0.001
Composite complication	1.31 (0.88–1.93); *p*=0.180	0.97 (0.63–1.49); *p*=0.874
Inhospital mortality	1.94 (0.58–6.51); *p*=0.281	1.74 (0.49–6.18);

Results are shown as odds ratio (95% CI); patients without preoperative anaemia were used as the reference; OR_unadj_ = unadjusted odds ratio; OR_adj−1_ = odds ratio; LOS = length of hospital stay.

**Table 5:** Postoperative complications.

Parameter	Total (*N* = 893)	Preoperative anaemia (*n* = 485)	No preoperative anaemia (*n* = 408)	*p* value
*Complications before discharge*				0.101
Pain at incision site	76 (8.5%)	42 (8.7%)	34 (8.3%)	—
Surgical site infection	22 (2.5%)	17 (3.5%)	5 (1.2%)	—
Difficulty in breathing	5 (0.6%)	3 (0.6%)	2 (0.5%)	—
Fever	7 (0.8)	2 (0.4%)	5 (1.2%)	—
Palpitations	4 (0.4%)	4 (0.8%)	0 (0.0)	—
Others	6 (0.7%)	4 (0.8%)	2 (0.5%)	—

Results are shown as frequency (percentage); others = hypoventilation and cardiogenic shock.

**Table 6:** Effects of red blood cell transfusion on outcome.

Logistic regression analyses (adjusted)	OR_unadj_ (95% CI); *p* value	OR_adj−1_ (95% CI); *p* value
P-LOS association with RBC transfusion	3.81 (2.64–5.50); *p* ≤ 0.001	4.48 (2.67–7.51), *p* ≤ 0.001
Composite complication association with RBC transfusion	1.73 (1.11–2.69); *p*=0.015	1.32 (0.74–2.36); *p*=0.347
Inhospital mortality association with RBC transfusion	5.80 (1.82–18.53); *p*=0.004	0.52 (0.19–1.46); *p*=0.217

Results are shown as odds ratio (95% CI); no RBC transfusion was used as a reference; OR_adj−1_ = odds ratio adjusted for severity of anaemia, age, sex, comorbid conditions, surgical specialty, grade of surgery, and cancer; CI = confidence interval; P-LOS = prolonged length of hospital stay; RBC = red blood cell.

**Table 7:** Perioperative use of haematinics in the patients.

Parameter	Patients total (*n*)	Oral iron	Folate	Oral iron + folate	Others
*Perioperative*	893	74 (8.3%)	29 (3.2%)	38 (4.3%)	40 (4.5%)
*Preoperative haematinics*					
Preoperative anaemia^a^	485	28 (5.8%)	7 (1.4%)	9 (1.9%)	14 (2.9%)
No preop. anaemia^a^	408	2 (0.5%)	1 (0.2%)	0.0%	2 (0.5%)
*Postoperative haematinics*					
Preoperative anaemia^a^	485	47 (9.7%)	26 (5.4%)	39 (8.0%)	23 (4.7%)
No preop. anaemia^a^	408	7 (1.7%)	5 (1.2%)	5 (1.2%)	17 (4.2%)

Results are presented as percentages; ^a^ more than one treatment per patient is possible, that is, some patients received a combination of pre- and/or posthaematinics treatments; others = oral iron and/or folate administered with vitamin B_12_, erythropoietin, or zinc; no patient received haematinics intraoperatively.

**Table 8:** Analysis of excluded data.

Parameter	Total∗ (*N* = 60)	PA (*n* = 41)	No PA (*n* = 18)	*p* value
*Age, mean (range)*	44.2 (16–90)	47.0 (15–86)	46.0 (16–83)	0.027
*Sex, n (%)*				0.347
Females	21 (35.6%)	13 (31.7%)	8 (44.4%)	—
Males	38 (64.4%)	28 (68.3%)	10 (55.6%)	—
*Preoperative Hb concentration*				
Mean Hb ± SD	11.2 ± 2.5 g/dl	9.9 ± 1.8 g/dl	14.0 ± 1.1 g/dl	<0.001
Mild anaemia	—	13 (21.0%)	—	—
Moderate anaemia	—	24 (40.7%)	—	—
Severe anaemia	—	4 (6.8%)	—	—
*Surgical specialty, n (%)*				0.670
General surgery	33 (55.9%)	23 (56.1%)	10 (55.6%)	—
Gynaecology	1 (1.7%)	1 (2.4%)	0 (0.0%)	—
Orthopaedics	16 (27.1%)	13 (31.7%)	5 (27.8%)	—
Urology	4 (6.8%)	3 (7.3%)	1 (5.6%)	—
Plastics	2 (3.4%)	1 (2.4%)	1 (5.6%)	—
Ear, nose, and throat (ENT)	24 (2.7%)	13 (2.7%)	11 (2.7%)	—
Neurosurgery	1 (1.7%)	0 (0.0%)	1 (5.6%)	—
*Sickling status (n = 24)*				0.746
Positive	8 (20.8%)	3 (16.7%)	2 (33.3%)	—
Negative	19 (79.2%)	15 (83.3%)	4 (66.7%)	—
*Comorbidity (n = 54)*				0.245
Yes	15 (27.8%)	13 (33.3%)	2 (13.3%)	—
No	39 (72.2%)	26 (66.7%)	13 (86.7%)	—
*LOS mean ± SD (days) (n = 49)*	33.3 ± 17.8	35.8 ± 17.0	27.7 ± 19.0	0.632
*Mortality (n = 54)*				0.675
Yes	2 (3.7%)	2 (5.3%)	0 (0.0%)	—
*Complications (n = 54)*				0.797
Yes	21 (38.9%)	14 (36.8%)	7 (43.8%)	—
*Perioperative transfusion (n = 55)*				0.499
Yes	19 (34.5)	15 (39.5%)	4 (23.5%)	—

∗One patient had a high Hb level; hence, that patient was not included in the analysis; 59 samples were analysed; LOS = length of hospital stay, PA = preoperative anaemia.

## Abbreviations

Hb: Haemoglobin
RBC: Red blood cells
MCV: Mean corpuscular volume
MCH: Mean corpuscular haemoglobin.
